# Microhotplates for Metal Oxide Semiconductor Gas Sensor Applications—Towards the CMOS-MEMS Monolithic Approach

**DOI:** 10.3390/mi9110557

**Published:** 2018-10-29

**Authors:** Haotian Liu, Li Zhang, King Ho Holden Li, Ooi Kiang Tan

**Affiliations:** 1 School of Mechanical and Aerospace Engineering, Nanyang Technological University, Singapore 639798, Singapore; liuht@ntu.edu.sg; 2 Temasek Laboratories, Nanyang Technological University, Singapore 67905910, Singapore; li_zhang@ntu.edu.sg; 3 School of Electrical and Electronic Engineering, Nanyang Technological University, Singapore 67905367, Singapore; eoktan@ntu.edu.sg

**Keywords:** gas sensor, metal oxide (MOX) sensor, micro-electro-mechanical system (MEMS), microhotplate

## Abstract

The recent development of the Internet of Things (IoT) in healthcare and indoor air quality monitoring expands the market for miniaturized gas sensors. Metal oxide gas sensors based on microhotplates fabricated with micro-electro-mechanical system (MEMS) technology dominate the market due to their balance in performance and cost. Integrating sensors with signal conditioning circuits on a single chip can significantly reduce the noise and package size. However, the fabrication process of MEMS sensors must be compatible with the complementary metal oxide semiconductor (CMOS) circuits, which imposes restrictions on the materials and design. In this paper, the sensing mechanism, design and operation of these sensors are reviewed, with focuses on the approaches towards performance improvement and CMOS compatibility.

## 1. Introduction

Gas sensors have been widely applied in various fields, such as agriculture [1], automotive [2], industrial, indoor air quality monitoring [3] and environmental monitoring [4,5]. Recently, the prevalence of the Internet of Things (IoT) stimulates the development of sensors with small sizes (<10 mm × 10 mm × 10 mm) [6]. In addition, miniaturization of the gas sensors drives the development of electronic noses (E-nose) in various fields, such as food quality control [7,8], disease diagnosis [9,10] and indoor air contaminants classification [11]. Micro-electro-mechanical systems (MEMS) technology is crucial to design and fabricate miniaturized gas sensors with excellent performance such as low power consumption (<100 mW), high sensitivity and fast response/recovery [12]. Additional benefits come from the low cost of the sensor from batch fabrication and the potential to integrate them with signal conditioning circuits.

**Figure 1:**
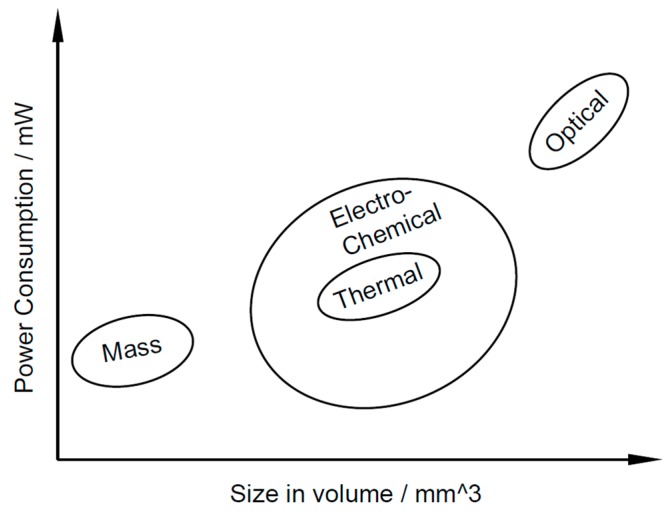
Comparison of the size and power consumption of the four types of gas sensors.

The gas sensors can be categorized into four types according to Janata [13]: (1) mass sensors; (2) optical sensors; (3) thermal sensors; and (4) electrochemical sensors. A comparison of these four types of sensors is summarized in Table 1, and their relative sizes and power consumptions are shown in Figure 1.

The mass sensors measure the frequency shift of the acoustic wave when foreign particles or molecules are absorbed onto the surface of an oscillating structure. Gravimetric sensors, such as quartz crystal microbalances (QCM), surface acoustic wave (SAW) sensors, surface transverse wave (STW) sensors and shear-horizontal acoustic plate mode (SH-APM) sensors, use quartz crystals with different cuts as the oscillating structure. They are extremely sensitive, with resolutions down to nanogram level. The frequency shift was observed to have an approximately linear relation with the concentration of the gas by Öztürk et al.; additionally, the sensitivity of their QCM sensor can reach a few hertz per ppm [14]. However, the acoustic wave properties heavily depend on the temperature, which gives rise to environment requirements for their applications. Moreover, quartz is not compatible with complementary metal oxide semiconductor (CMOS) processes, and thus, monolithic integration remains challenging.

Thermal sensors detect the gas by the temperature-induced resistance change when combustion reactions take place in catalytic materials. The sensors are low cost and reliable in dusty environments; hence, they are widely used in industrial applications. However, catalytic based reactions restrict the applications of these sensors to flammable gases such as hydrogen, methane, propane and butane. Moreover, catalytic poisoning may deteriorate the long-term stability of the sensors.

Optical sensors recognize the gas by absorption of light with certain frequencies. These frequencies are closely related to the oscillation of molecules; the spectrum suggests that the bonds exist in the molecules. Optical sensors are highly selective and have much higher cost than the other three types because of the complex interferometer mechanisms and the light source. The interferometer and light source also impose challenges on their miniaturization and system integration. Therefore, the high cost and power consumption limit their on-site applications, and most optical sensors still remain as laboratory apparatus [15].

Electrochemical sensors are based on the change in electrical properties when target gas diffuses and reacts with the sensing material. Electrochemical sensors are relatively easy to be miniaturized and manufactured with microfabrication technologies. Successful miniaturizations include field effect transistor (FET) gas sensors and metal oxide semiconductor (MOX). FET sensors are based on the change of the threshold voltage when gas molecules reach the gate material. It is fully compatible with CMOS processes and has a sub-milliwatt level power consumption. However, metal gate materials, such as palladium and platinum, are only sensitive to hydrogen. Recently, many works have been done on the gate design to improve the performance of the FET sensors by applying nanomaterials, such as carbon nanotubes [16] and graphene [17], or by a thin charge inversion layer [18]. MOX gas sensors measure the conductance change of the metal oxide layer and play a dominant role in both research studies and commercial products because they have the most balanced overall performance and low fabrication cost.

Companies such as ams AG [19], Bosch Sensortec [20], Figaro [21] and Sensirion [22] have developed successful commercial MEMS MOX gas sensors for indoor air quality management to detect volatile organic compounds (VOCs). The specifications of some of their products are listed in Table 2. The size of the system depends not only on the hotplate, but also on the package and supporting components. Packaging technology is beyond the scope of this review.

The demand for continuous miniaturization and power consumption reduction drive the research of MOX gas sensors towards direct integration of the MEMS sensing structure with the integrated circuits for signal conditioning circuits [23]. This integration is conventionally realized with the multi-chip approach in which the sensor and circuits are designed and fabricated on separate chips. Multi-chip integration enables independent optimization of the MEMS sensor and CMOS circuits. It also provides more flexibility in the design and fabrication, so that less development time is required. However, extra cost is incurred by the complex packaging and wire bonds. The parasitic element of interconnections gives rise to additional noise. A more advanced way for CMOS-MEMS integration is the monolithic approach, where the sensor and circuits are designed and fabricated on a single substrate. The monolithic integration enhances the performance of the sensor by reducing its size, power consumption and noise [24]. The high development cost and long development cycle can be offset by reduced packaging cost for large-volume manufacturing. The challenge of the monolithic approach is the limitation of materials available for the CMOS processes [25], which will be discussed in detail in this review. 

Different aspects of the CMOS-MEMS integration technology have been reviewed by many researchers. For example, Qu focused on the fabrication technologies [26], Li et al. focused on electrochemical biosensors [27], Fischer et al. focused on the integration approaches of MEMS and integrated circuits (IC) and Hierlemann et al. reviewed the fabrication techniques of all the four types of chemical sensors [28]. This review specifically focuses on the recent development in metal oxide CMOS-MEMS gas sensors based on a monolithic approach. The sensing mechanism of metal oxide will be introduced first to define the parameters used to evaluate the performance of the sensor and understand the necessity of the microhotplate in the sensor. The design concerns of the microhotplate are then discussed, with an emphasis on selecting CMOS compatible materials and achieving lower power consumption. Next, the characteristics and synthesis methods of sensing material are briefly discussed. Last, the challenges for integration of the circuit and the sensor are explored.

## 2. Sensing Mechanism

The reaction between the target gas and the metal oxide film is composed of the reaction of the target gas with metal oxide molecules, as well as the preabsorbed oxygen species. The sensing process consists of three steps: diffusion of the target gas molecules onto the surface of the metal oxide, adsorption of the gas molecules into the metal oxide and reaction between the gas and metal oxide. 

In the case of n-type semiconductor metal oxides, the sensing mechanism can be explained by the double Schottky barrier model [29]. The interaction between the metal oxide and the oxygen molecules in air or other oxygen-containing environment generates different oxygen species, such as $O_{2}\left(\alpha_{1}\right),{\;O}_{2}^{-}\left(\alpha_{2}\right),{\;O}^{-}\left(\beta \right)$ ${\mathrm{and}\;O}^{2-}\left(\gamma \right)$ (Equations (1)–(3)):

(1) $$\;O_{2}+e^{-}⇌{\;O}_{2}^{-}\;$$

(2) $$\;O_{2}^{-}+e^{-}⇌2O^{-}\;$$

(3) $$\;O^{-}+e^{-}⇌{\;O}^{2-}\;$$

The released electrons are trapped in the conduction band, forming depletion layers on the surface of the grains. The double Schottky barrier and upward bending of the conduction band at the grain boundaries lead to an increase in the resistance of metal oxide film. When a reducing agent, such as hydrogen, carbon monoxide or ethanol, is brought into contact with the metal oxide film, the resistance drops because of the neutralization of the oxygen species and mitigation of potential barriers. Conversely, an oxidizing agent, such as NO_2_, competes with the oxygen species for the electrons and further increases the resistance of the film.

The response of the sensor is defined by the ratio of the resistance of the sensing material before and after exposure to the target gas (Equations (4) and (5)) [29], where R_a_ and R_g_ are the resistance of the thin film before and after exposure to the target gas, respectively. Sometimes, the net change in resistance replaces the numerator.

(4) $$S=\frac{R_{a}}{R_{g}},\;\mathrm{for}\;n-\mathrm{type}\;\mathrm{semiconductor}$$

(5) $$S=\frac{R_{g}}{R_{a}},\;\mathrm{for}\;p-\mathrm{type}\;\mathrm{semiconductor}\;$$

The reaction rate between the chemical species and metal oxide film governs the response of the gas sensor. The α and β species dominate the temperature below and above 300 °C, respectively, while the γ type starts to appear from 550 °C onwards [30]. The reactive species are relatively unstable if the temperature is further increased and the dissolved oxygen atoms compete with the target gas for available active sites. In addition, a further increase in the temperature causes desorption of absorbed gas, causing the sensor response to be further reduced. Therefore, the response of a gas sensor versus the temperature behaves like a bell-shaped curve, with the maximum response obtained at the optimum operating temperature. This optimum temperature varies with respect to the types of metal oxides and the target gas. Therefore, gas sensors usually contain a heating element and an external or internal temperature system to ensure the optimal performance of the sensor.

**Figure 2:**
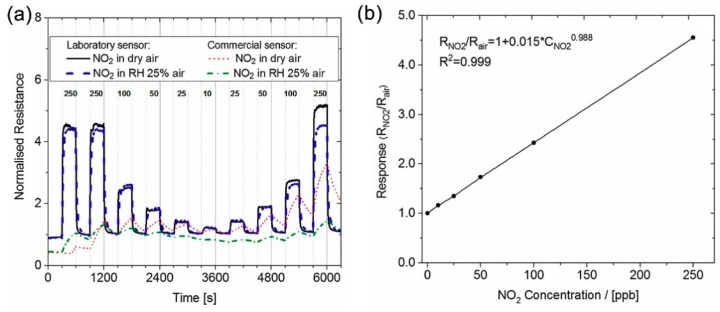
(**a**) The response of a laboratory WO_3_ based gas sensor and commercial MOX sensor with respect to NO_2_ in dry and humid air (**b**) response of the laboratory sensor as a function of NO_2_ concentration (from [33]).

The response has to be closely related to the centration of gas for effective detection. At a fixed temperature, the sensor response increases with respect to the concentration of the gas due to an increase in carrier concentration and mobility, which can empirically be represented by the power law (Equation (6)) [31]:

S = A_g_∙P_g_^m^ (6)

where A_g_ and m are constants and P_g_ is the partial pressure of the gas, which is proportional to the concentration of the gas. The value of m depends on the concentration of oxygen species when the metal oxide is exposed to air and is specific to each pair of target gas and metal oxide. The value of m can be approximated by theoretical approaches. Recently, Hua et al. proposed reduced receptor and transducer functions to calculate the value of m [32]. Figure 2a,b show a typical response of WO_3_ gas sensor to NO_2_ [33]. The effects of concentration on the response of the sensor can be clearly observed.

Additionally, the sensor response has a strong dependency on the grain size of the metal oxide crystal. Xu et al. [34] found that the resistance of the SnO_2_ gas sensor in response to reducing gas would decrease sharply when the grain size fell under two times of the width of the depletion layer, since electron transport was suppressed due to fully depleted grain. Rothschild and Komen showed from a numerical simulation that the sensitivity is proportional to the inverse of the grain size [35]. Yamazoe and Shimanoe proposed the volume and regional depletion concept to formulate the response for small crystals and crystals with planar, spherical and cylindrical shapes [36]. 

## 3. MEMS Microhotplate

### 3.1. Device Layers and Design Considerations

**Figure 3:**
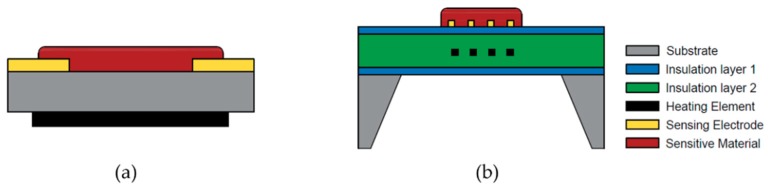
Schematics of (**a**) a traditional metal oxide gas sensor and (**b**) a microhotplate metal oxide gas sensor.

The device structures of a traditional metal oxide gas sensor and a MEMS-based metal oxide gas sensor are shown in Figure 3a,b, respectively. A metal oxide semiconductor gas sensor usually consists of three main components: the microhotplate for temperature elevation, the sensitive material for gas detection and the electrodes for signal transmission. The microhotplate includes a substrate layer, an insulation layer, a heater layer and a passivation layer. The detailed layers are listed as follows:

- The substrate.
- The bottom silicon oxide/nitride layer, which insulates the heating element from the substrate.
- The heating element layer and an adhesion layer, if necessary. Sometimes, temperature sensing elements, such as resistance temperature detectors (RTD), are also fabricated on this layer to monitor the temperature of the microhotplate and provide a reference in the temperature control loop.
- The top silicon nitride/oxide layer, which serves as the insulation between the heater and the sensing material or electrode and passivation layer to prevent catalytic interaction between the target gas and the heater material [37].
- The electrode layer.
- The sensing material layer.

The performance of a sensor is usually evaluated by its 4S, i.e., sensitivity, speed, stability and selectivity. In gas sensor applications, the sensitivity is expressed by the response and detection range. The speed is evaluated by the response and recovery time, which is defined as the time taken to achieve 90% of the change in the resistance. In addition to the 4S, the power consumption of the sensor needs to be minimized due to the voltage and current limit for miniaturized sensors. Extensive studies have been conducted to improve the design of each component for the optimized performance; these will be reviewed accordingly in this section.

The function of the microhotplate is to raise the temperature of the sensitive material to its optimum operating temperature and it is the main source of power consumption in the gas sensor. Bhattacharyya [38] and Spruit [39] have given excellent reviews on the design of microhotplates, concerning the power consumption, temperature homogeneity and mechanical stability.

### 3.2. Microhotplate

#### 3.2.1. Substrate

Silicon is the mainstream substrate material for micro gas sensors due to its capability with IC fabrication processes and the potential for CMOS integration as a monolithic system for in-situ sensing and processing. Non-silicon materials, such as ceramics (alumina, zirconia), borosilicate glass [40], silicon carbide [41] and plastic [42], are also explored for applications in harsh conditions. These sensors are beyond the scope of this review due to the technological limitations to integrate them with the traditional CMOS circuits at the current stage. The substrate material of all sensors discussed in the following sections of this review is silicon.

#### 3.2.2. Microhotplate Configurations to Reduce Power Consumption

The majority of the power consumed by the microhotplate is converted into thermal energy and dissipated into the surroundings. Therefore, it is essential to suppress the heat loss from the various heat transfer processes, i.e., the conduction from the sensing area to the substrate, the conduction from sensing area to the air, the convection from the sensing area to air and the radiation to the environment. The amount of heat transfer can be obtained from the Equations (7)–(9) [43]:

(7) $$Q_{\mathrm{cond}\;}=-{\mathrm{kA}}_{\mathrm{cond}}\nabla T\;$$

(8) $$\;Q_{\mathrm{conv}}{\;=\;\mathrm{hA}}_{\mathrm{conv}}{(T}_{s}-T_{a})\;$$

(9) $$Q_{\mathrm{rad}}{\;=\;\varepsilon A}_{\mathrm{rad}}{\sigma (T}_{s}^{4}-T_{b}^{4})\;$$

where k is the thermal conductivity of the film material; h is the coefficient of convection of air; ε is the emissivity of the microhotplate; σ is the Stefan-Boltzmann constant; A_cond_, A_conv_ and A_rad_ are the areas of the surfaces where each heat transfer process takes place; ∇T is the temperature gradient within the solid; T_s_ is the surface temperature; T_a_ is the ambient temperature; and T_b_ is the temperature of the surrounding material, such as the substrate or the package. From Equations (7)–(9), the power consumption can only be reduced by reducing the operating temperature or the area of heat loss. Reduction in the operating temperature can be achieved by depositing metal oxide films with nanostructures, whose response can be compensated by the surface area to volume ratio. Nanostructures and their synthesis method will be discussed in Section 3.4. The latter relies on removing as much material between the heat source and sink as possible and corresponds to the approaches of temperature isolation. Therefore, given the same sensing material, the power consumption can only be minimized by area reduction.

**Figure 4:**
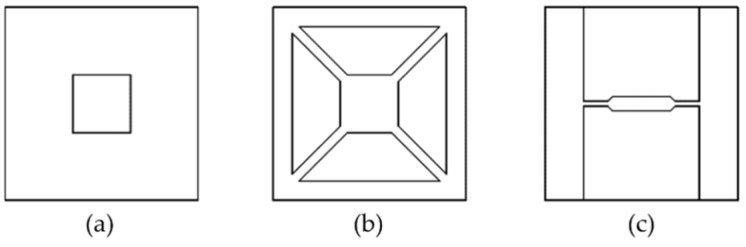
Top view of microhotplates with different configurations: (**a**) closed membrane; (**b**) suspended membrane; (**c**) bridge.

Conduction and convection are the main heat loss mechanisms. Although some studies treated conduction as the primary mechanism [44,45,46], it is suppressed in microhotplates because of the thin features. Heat loss through convection is comparable and may contribute to up to 60% of the total heat loss in some cases [47,48]. The area in Equation (7) refers to the contact area between the high-temperature zone and the rest of the microhotplate; hence, power consumption can be reduced by geometry optimization. Closed membrane, suspended membrane and bridge (Figure 4a–c, respectively) are the main configurations adopted in previous studies. The substrate right underneath the sensing region is etched away in all three configurations, eliminating the largest contact area. Air acts as heat insulation material due to its much lower thermal conductivity than silicon (0.018 Wm^−1^ K^−1^ compared to 145 Wm^−1^ K^−1^ for silicon). 

**Figure 5:**
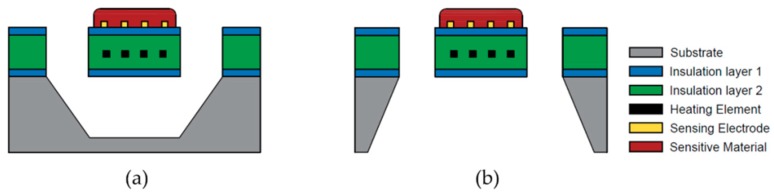
The suspended membrane microhotplate structure formed by (**a**) front-side etching and (**b**) back-side etching.

At the expense of reduced power consumption, the mechanical structure of the microhotplate becomes more fragile due to less supporting materials; thus, the configuration must be designed according to the applications. Closed membranes are preferred for commercialized products or on-site applications since the sensors need to withstand vibrations. In research on the other hand, suspended membranes are more popular, since it is able to push the power consumption performance to a limit. In addition, its low thermal inertia contributes to fast response and recovery time and enables reliable temperature modulation. The suspended membrane can either be released from the front side (Figure 5a) or the back side (Figure 5b). Front-side etching requires additional protection of the structure layer or sacrificial layer to create the cavity. Therefore, back-side etching is more common for the sake of simplicity of the processes. Efforts are made to improve the mechanical stability of the suspended structure. Iwata et al. [49] has proposed to reinforce the bridges with the thick polymer layer SU-8 due to its extremely low thermal conductivity ($0.2{\;\mathrm{Wm}}^{-1}K^{-1}$) compared to silicon, as well as its compatibility with micromachining processes. However, the reinforced layer increases the fabrication complexity, which is why it has not been adopted in other studies so far. The bridge configuration is an extreme case of the suspended membrane. It further reduces the area for conduction by leaving only two bridges to suspend the hotplate. However, it suffers more from the mechanical instability, as well as the smaller sensing area due to the shrunk hotplate; hence, it is not widely applicable in research works and commercial products.

Both of the areas in Equations (8) and (9) refer to contact area between the microhotplate and air. This area is usually predetermined by the overall size of the device; hence, not much improvement can be made during the design. The convection heat loss to the air can be obtained by fluid simulations [50]. This is ignored in most studies because it only takes up a negligible percentage. Radiation loss soars with the fourth power of the temperature in Kelvin. Pike and Gardner claimed that convection contributes to less than 10% of the total heat loss for applications below 500, according to their lumped element model [51]. Mele et al. claimed from the same model that the radiative loss needs to be taken into account when the temperature reached 600 °C, and is more significant than conductive loss for temperatures above 1000 °C. 

**Figure 6:**
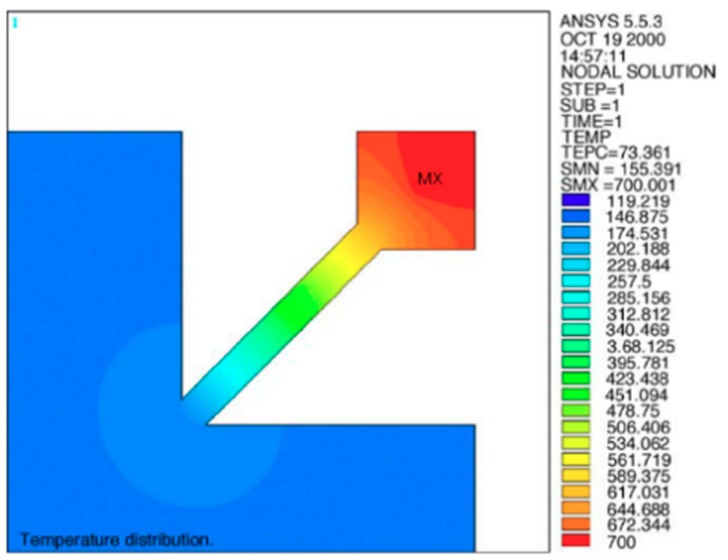
Temperature distribution of a suspended membrane microhotplate without gas sensitive layer (from [54]).

A Finite Element Method (FEM) electro-thermal simulation is commonly implemented during the microhotplate design process for optimization of the temperature distribution and power consumption. A large variety of commercially available software such as ANSYS [49,52,53,54,55,56], COMSOL Multiphysics [57,58,59,60,61,62,63], ConventorWare [64,65], ISE TCAD [66,67,68] and MEMCAD [69] has been used. Figure 6 shows the temperature distribution of a suspended membrane microhotplate without a gas sensitive layer for a target temperature of 700 °C [54]. Note that only a quarter of the device was used, as the geometry and symmetry boundary conditions were applied to improve the efficiency of the simulation. In addition to the temperature distribution and uniformity, numerical simulation can also provide critical information for structure design such as the vertical displacement and the maximum stress. However, accurate results are difficult to obtain due to limited access to the properties of thin films and simplifications made in these simulations. The thermal conductivities of the oxide, nitride and metal layers are often directly extracted from open literature. However, the real values vary between different foundries, depending on the conditions of the deposition processes. In addition to the structure layer, introducing the sensing layer makes the thermal analysis of the microhotplate more complicated. The anisotropy of the nanostructures makes its properties hard to be characterized. Moreover, this layer cannot be simply ignored, because the high thermal conductivity of the metal oxide contributes to the temperature uniformity [70]. Therefore, an on-site measurement of thermal conductivity is required; however, no group has done it so far to the best of the author’s knowledge.

The temperature profile of the optimized geometry has to be validated by the measurements of an infrared radiation (IR) camera or IR pyrometer. The relationship between the power consumption and the temperature of the hotplate can be obtained by the thermal image together with the measured voltage and current.

The microhotplate is a sandwiched structure formed by the insulation layer, the heater layer and the passivation layer to support the sensing material. Concerns in high-temperature stability determine the material and processes of the sandwiched layers. Residual stress and mismatch in thermal expansion coefficient induce a vertical displacement of the microhotplate at high temperatures and may lead to delamination [48]. Large displacement or weak adhesion might lead to structural failure. Rao et al. reported that the plasma-enhanced chemical vapor deposition (PECVD) nitride layer would peel off easily at a temperature above 691 °C, and thus, the low pressure chemical vapor deposition (LPCVD) nitride layer was adopted instead in their molybdenum hotplate [71]. Low-stress nitride (Si_x_N_y_) or oxide/nitride/oxide (ONO) layer [63,72,73,74] is preferred for their low residual stress and good adhesion to metal. Except for nitride and oxide, silicon carbide is also explored as the alternative passivation material by Saxena et al. [75]. A reduced thickness of the material is required to achieve similar thermal behavior, however, the nitride layer is still preferred because of the low cost and simplicity of the CMOS processes.

#### 3.2.3. Heater Material

The heating element material has to be stable at high temperature and is preferred to have a linear relationship (Equation (10)) between its resistance and temperature within the operation range:

R = R_0_ (1+ α(T − T_0_)) (10)

where T_0_ is the baseline temperature, R_0_ is the resistance at the baseline temperature, α is the temperature coefficient of resistance T is the operating temperature and R is the resistance at the operating temperature. The linear behavior assisted the measurement and control of temperature and simplified the signal processing steps. The temperature measurement was either performed by the heater itself or a thermometer on the same plane.

Chemical and mechanical stability at high temperatures were the main concerns for heater material selection. Platinum heaters are the most extensively studied material due to its high thermal conductivity and stability up to 600 °C, which is above the optimum operating temperature of most metal oxides. A layer of titanium or tantalum with tens of nanometers thickness is usually sandwiched between the platinum and insulation layer for better adhesion. For operations above 500 °C, titanium is not recommended, because it will diffuse into the platinum layer and form precipitates on the grain boundaries [76], whereas tantalum shows better behavior due to its function as a diffusion barrier [77,78]. Ceramic adhesion films have also been investigated. Ababneh et al. reported that titanium dioxide adhesion layer would not diffuse into platinum for temperatures up to 800 °C [76]. Halder et al. obtained stable performance of electrodes on a platinized substrate at 1000 °C, with aluminum oxide as the adhesion layer, due to an increase in grain size [79]. Although TiO_2_ and Al_2_O_3_ show much better adhesion performance, their applications are restricted by the feasibility of integration into the CMOS process due to their high-temperature deposition conditions. In addition to single layer adhesion films, a Cr/CrN/Pt/CrN/Cr multilayer approach was demonstrated by Chang and Hsihe [80], which shows improved adhesion and structural stability up to 480 °C. However, the multilayer approach has not been widely applied, because it introduces extra depositing and etching steps to the fabrication processes. 

Doped polysilicon is another widely adopted heater material with linear resistance-temperature relations. It is a fully CMOS-compatible material and the adhesion problem no long exists. However, polysilicon is only suitable for operating temperatures below 500 °C, beyond which the recrystallization of polysilicon will cause drift in resistance [81]. Special packaging techniques, such as inert gas sealing, are required to alleviate this problem [82], which are not preferred for commercialization due to its higher cost.

For microhotplates operating above the stability point of platinum and polysilicon, molybdenum [47,71] and tungsten [50,83,84] heaters were studied for applications in harsh conditions. Other than these two metals, Creemer et al. investigated microhotplates based on CMOS compatible titanium nitride (TiN) heater and operating temperature can reach up to 700 °C but the high stress of TiN decrease the yield of the device [48]. The materials above are relatively high cost and mainly used in applications above 300 °C. More affordable materials, such as nickel, serve as better alternatives for operating temperature below 300 °C [55]. Table 3 summarizes the maximum temperature and CMOS compatibility of the various heater materials. Doped polysilicon and tungsten are preferred for monolithic CMOS-MEMS devices due to their compatibility with the processes and their temperature range can cover most operating temperature of the metal oxides. 

#### 3.2.4. Heater Geometry to Improve Temperature Uniformity

Both electro- and thermo-migration of the material could cause the degradation of the heater [87]. The former cannot be avoided due to the high operating temperature while the latter depends on the local temperature gradient. Therefore, maintaining the temperature uniformity across the microhotplate is the key to ensure the stable performance of the sensor over its lifetime. 

**Figure 7:**
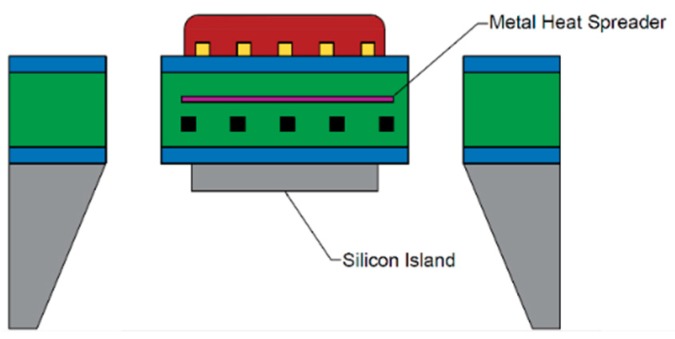
Silicon island and metal heat spreader application on a microhotplate.

Temperature uniformity can be improved by attaching a layer of material with high thermal conductivity to the microhotplate. The high thermal conductivity material can either be a silicon island [69] left after back side etching or a metal heat spreader plate [49,88] deposited above the heating element (Figure 7).

**Figure 8:**
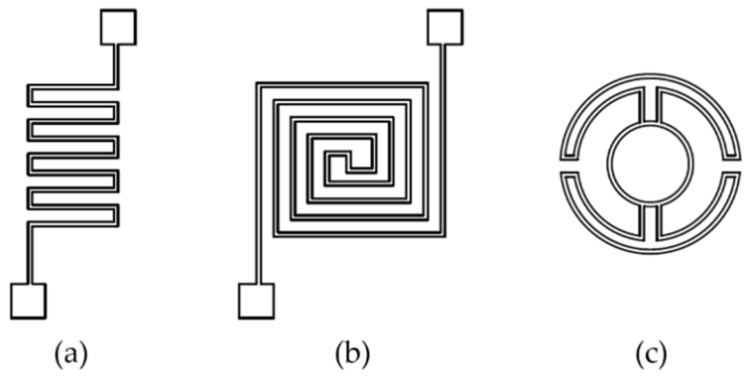
Heater geometries (**a**) meander, (**b**) double spiral, (**c**) drive wheel.

Alternatively, optimization of heater geometry with simulation and validation experiments has a significant impact on the temperature uniformity across the microhotplate. Meander, double spiral and drive-wheel are the main geometries reported (Figure 8). Meander shape is, thus far, the most extensively studied one due to its simple geometry. Lee et al. reported that hotspots formation at the center of the meander heater may lead to large temperature variations across the plate [67]. Their numerical simulations and experiments showed remarkably improved temperature uniformity of the drive-wheel type compared to other designs, such as the ultra-low resistance and the honeycomb design.

**Figure 9:**
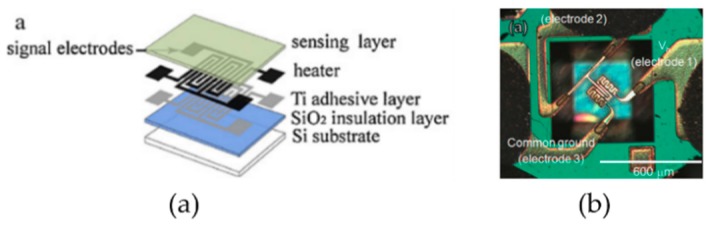
Coplanar geometry where heating and sensing electrodes lie on the same plane: (**a**) Au-loaded In_2_O_3_ nanofiber gas sensor by Xu et al. (from [89]) and (**b**) SnO_2_ nanowire gas sensor by Hwang et al. (from [90]).

Except for the sandwiched structure of the microheater, insulation and electrode layers, the heater and electrode can be fabricated on the same plane provided the same material is used (Figure 9a,b). This co-planar design reduces the number of lithography, patterning and etching steps required during the fabrication process. The coplanar heater can also eliminate the parasite capacitance formed between the heater and the sensing layer in sandwiched design [89]. Hwang et al. claimed that coplanar configuration is also advantageous for effective and rapid diffusion. 

Besides heater electrode deposited on a planar surface, a microhotplate with three-dimensional heater deposited on a three-dimensional V-shape groove was developed for a catalytic type gas sensor [91]. The heating elements lied on a concave surface to obtain a larger active area. However, no metal oxide gas sensor has been developed on the three-dimensional heater thus far, possibly due to the non-uniform temperature distribution across the active area.

### 3.3. Electrode

The electrode transmits the resistance of the metal oxide film to the signal conditioning circuit during operation: its material is required to have high electrical conductivity, high-temperature stability, good adhesion to the passivation layer and low contact resistance [37]. In addition, the backside/frontside etching to release the membrane is performed after the deposition of the electrode material. Therefore, the electrode material should be inert to the etchant; otherwise, a protection layer is required before the etching process. Most of the electrodes for the MEMS gas sensors are noble metals with an adhesion layer such as Au/Ti, Au/Cr or Pt/Ti. The performance of the electrode will drift after long time operation. Capone et al. observed the degradation of Au/Ti electrodes due to diffusion after the continuous operation at 300 °C and 600 °C. They suggested applying artificial neural network for signal processing to reduce the drift [92]. Noble metal electrodes are believed to have catalytic effects to improve the response of the sensor [93]; however, this point has not been proven yet. In CMOS-MEMS gas sensors, the gold or platinum electrodes are deposited in a post-CMOS process, because they are not CMOS compatible. Sometimes, aluminum is adopted for a full CMOS-compatible process. However, this faces a long-term stability issue under high-temperature operation. The electrode material is deposited in an interdigitated shape so that the large resistance of the metal oxide layer is measurable at low voltage and power consumption. A comprehensive review of the electrode material for both micro gas sensors and traditional gas sensors is given in [94]. 

### 3.4. Sensing Material

Eranna has reviewed various types of bulk metal oxide materials and their gas sensing applications [95]. Both n-type (WO_3_, SnO_2_, ZnO, TiO_2_, V_2_O_5_) and p-type (NiO, CuO, Co_3_O_4_,) metal oxide semiconductors have been investigated extensively as the sensing materials. However, the n-types takes up more than 90% of the work [96], since the mobility of charge carriers of n-type oxide semiconductors are higher than that of the p-types [30]. Table 4 summarizes the performance of some microhotplate MOX gas sensors.

As the gas sensing reactions occur through gas adsorption, charge transfer and desorption on the surface of the sensing materials, the sensing response is highly dependent on the amount of available surface active sites. In this regard, zero-dimensional nanoparticles with abundant active sites, due to increased specific surface area, are highly desirable for promoting improved gas adsorption and prompting more target gas molecules to participate in the oxidation and reduction reactions. Uniform distribution of nanoparticles with minimum agglomeration is essential to ensure good gas-sensing reactions for this type of material. One-dimensional (1D) nanostructured materials, such as nanowire [84,90,97], nanofiber [89], nanotube [98], nanobelts [99] and nanorods [100] are extensively studied for their high aspect ratio with increased surface-to-volume ratio, giving rise to better stabilities and superior gas-sensing sensitivity with fast response and recovery time. For these 1D nanostructured materials, a smaller grain size/diameter and longer length are, in general, more beneficial for improved gas sensing performance due to the availability of more active sites as the size shrinks and the improved electron transport along the axial direction. Other than these, a lot of research interests have been devoted to two-dimensional (2D) nanostructures for their unique morphologies. These 2D nanostructures, including nanosheets [101] and nanoplates [102], are usually coupled with porous nanostructures and network-like structures to better facilitate the penetration/adsorption of gas molecules into the sensing material, leading to an enhanced sensing response. More recently, three-dimensional (3D) hierarchical nanostructures are receiving increasing attention. These 3D nanostructures with flower-like, leaf-like and spindle-like morphologies are assembled from the low dimensional nanomaterials, including 0D nanoparticles, 1D nanorods and 2D nanosheets [103]. They are deemed to give rise to better sensing performance due to their large surface area, abundant active sites and fast interfacial transport than the low dimensional nanomaterials. Despite of the promising sensing performance, the challenges of inhomogeneity and poor reproducibility have to be addressed for more useful applications of these nanostructures. Noble metals are doped into the metal oxides as chemical or electronic sensitizers to improve the sensitivity, response/recovery time and selectivity of the gas sensor through the chemical or electronic sensitization mechanism [30]. In chemical sensitization, the noble metal assists the adsorption of the target gas by lowering the activation energy. The activated gas is then spilled over the surface of the grain. Most noble metal dopants fall into the electronic sensitization category. The metal particles and their oxidation states act as electrodes attracting the electrons, decreasing the free electrons in the conduction band and creating a depletion region [104]. The electrons return to the grain after exposure to reducing air. This leads to an increase in the resistance and the sensor response (R_a_/R_g_).

The concentration of noble metal has to be carefully controlled to avoid coagulation. A high percentage of doping may suppress the functionality of the sensor, since agglomeration may decrease the total area of the catalyst surface. Adding catalytic layer results in loss of sensitivity over a long period due to fouling or coking of catalytic metal when exposed to certain gas or vapors. Additionally, the stability of the sensor is affected by the degradation of the dopant metal. The high-temperature degradation of metal oxide materials is not limited by electromigration, but rather by outdiffusion and evaporation of dopants from bulk materials [105].

The metal oxide sensing layer is deposited via a post-processing process after the fabrication of the CMOS circuits and the MEMS microhotplate. Guo et al. have given a comprehensive review of the synthesis methods of metal oxide nanostructures [106]. The sensing material can be synthesized via chemical vapor deposition (CVD), physical vapor deposition (PVD), sol-gel, sintering, spray pyrolysis, spin casting, drop coating, screen printing and ink-jet printing. Among these approaches, CVD, PVD and sol gel are usually employed for thin-film deposition and are compatible with CMOS processes, while the others are more for thick-film with nanostructures. In general, sputtering is preferred over evaporation for its compatibility with a wider choice of materials, better step coverage and enhanced adhesion to the substrate. Surface functionalization or adding binders could further promote the surface adhesion between the sensing materials and substrates [95]. Alternative CMOS compatible ways have been explored. Annanouch et al. grew WO_3_/Cu_2_O nanoneedles directly on a MEMS hotplate by a single step aerosol-assisted CVD process [107]. The metal oxide nanostructure can also be grown with the internal microheater, whose grain size can be controlled by varying the temperature [108].

### 3.5. Fabrication Processes

**Figure 10:**
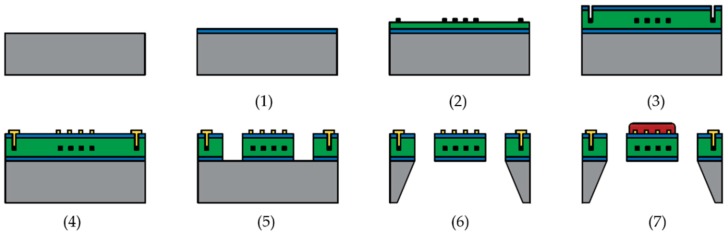
Fabrication processes of a suspended membrane microhotplate with backside etching.

Monolithic gas sensors were fabricated by a CMOS-first approach. The microhotplate materials are deposited and released after the CMOS circuits have been deposited into the wafer. The typical fabrication processes of a suspended membrane microhotplate with a metal heating element are listed as follows and shown in Figure 10:

1. Thermally grown/deposition of the insulation layer.
2. Lift-off/sputtering, patterning and etching of the heater material.
3. Deposition of the passivation layer; patterning and etching for electrical contact.
4. Lift-off/sputtering, patterning and etching of electrode material.
5. Patterning the oxide/nitride layer to define the geometry of the membrane and bridge.
6. Backside/frontside etching to release the suspended membrane.
7. Deposition of the metal oxide layer.

Both dry etching and wet etching have been used in the membrane step. Tetramethylammonium hydroxide (TMAH) or potassium hydroxide (KOH) can be used as the wet etchant, with the former preferred due to full compatibility with CMOS processes. Dry etching processes, such as deep reactive ion etching (DRIE), is, in general, more preferred because the vertical sidewall makes the footprint of the sensor smaller. 

**Figure 11:**
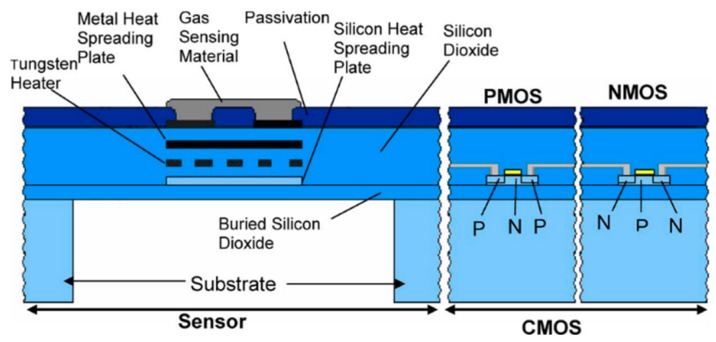
Monolithic gas sensor fabricated with silicon on insulator (SOI) CMOS technology (from [50]).

Despite the traditional layer-by-layer deposition approach, the silicon on insulator (SOI) CMOS technique was adopted to achieve simple fabrication, the high operating temperature of MOSFETs (400~600 °C), effective isolation and reduction in leakage current [50,66,88,109]. Either traditional metal heaters [53] or MOSFET heaters [66] can be used for temperature elevation. An example of SOI CMOS microhotplate is shown in Figure 11. The buried oxide layer acts as the etch stop layer during the membrane release step. Additionally, it insulates the heater from the silicon substrate. This technique makes monolithic integration of sensing and signals conditioning units of the system possible. 

## 4. Modes of Operation

The performance of the sensors listed in Table 4 is characterized under the isothermal condition, whereby the temperature and the applied voltage across the heating element remain constant throughout the operation. Low thermal inertia and fast thermal response allow the reduction of power consumption for micromachined gas sensors by pulse mode temperature modulation. In this mode, a square wave voltage is applied across the heater so that the heater is switched on and off. At the expense of reduced power consumption, the interval between two consecutive samplings is longer due to the low response in the low voltage region. For example, the iAQ-core P air quality sensor drops from 66 mW to 9 mW by applying temperature modulation, and the sampling time increases from 1 s to 11 s. Therefore, pulse mode temperature modulation is only suitable for situations when frequent data acquisition is not required. Courbat et al. achieved sub-milliwatt power consumption on their SnO_2_/3% Pd CO sensor [110]. Enhancement in the response of the ZnO-nanowire sensor was observed by Shao et al., when it was operating in pulsed mode [84]. They attributed this enhancement to the presence of high-temperature oxygen species during the low-temperature period. 

## 5. CMOS-MEMS Monolithic Gas Sensor

Although most microhotplates mentioned above are fabricated with CMOS compatible processes, few of them has actual CMOS circuits integrated for temperature control and signal conditioning. The main challenge for CMOS integration is that the operating temperature of the metal oxides (typically 250 to 400 °C) is usually higher than the maximum operating temperature of IC (<300 °C). Therefore, thermal isolation is crucial for monolithic gas sensor design. Suspended membrane geometry is preferred because of their excellent heat isolation performance. Less than 3 °C temperature difference between the circuit and ambient can be achieved by this geometry when the hotplate works at 400 °C [125].

**Figure 12:**
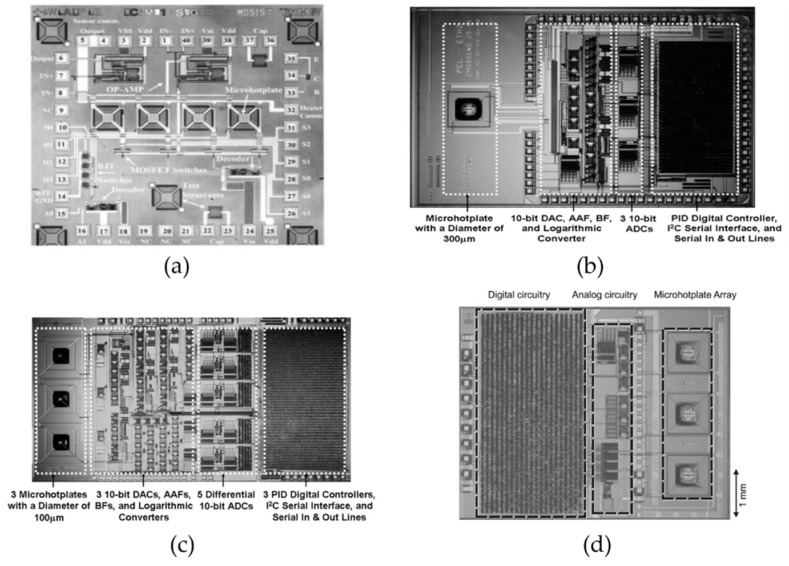
Monolithic CMOS-micro-electro-mechanical systems (MEMS) gas sensors: (**a**) microhotplate array for carbon monoxide detection (from [108]) (**b**) microhotplate gas sensor with proportional-integral-derivative (PID) controllers (from [125]), (**c**) microhotplate gas sensor with mixed-signal architecture (from [125]), (**d**) microhotplate gas sensor using biased current measurement (from [126]).

The signal conditioning circuit consists of the temperature control circuit, the resistance readout circuit and the serial bus interface for external communication. In the temperature control circuit, the heater is driven by the MOSFET switches [108] (Figure 12a) with the signal from digital-to-analog converters (DACs). The temperature is controlled by switching the heater on and off alternatively for different time intervals determined by the controller. The controller is of proportional type or proportional-integral-derivative (PID) type to reduce the steady-state error in the temperature control. The actual temperature becomes an input in the feedback loop and is compared with the target temperature. Temperature measurement is done by the on-chip thermometers and the result needs to pass through amplifiers and analog-to-digital converters (ADCs) before processing. The resistance measurement circuit also contains amplifiers and ADCs. In addition, it needs to deal with the large range of the resistance of the metal oxide film from KΩ to MΩ. The resistance signal has to be further processed so that it can be transferred within the allowable number of bits of the converters. The resistance can either be processed by a logarithmic converter [125] (Figure 12b,c) or measured with a biased current [126] (Figure 12d). The recognition of the type of the gas is done by external processors, such as microcontrollers or personal computers. Inter-integrated circuit (I^2^C) serial protocol is commonly adopted for external communication in both commercialized sensors and the research works because it can reduce the number of pins required. 

## 6. Future Trends and Challenges

It can be observed from Table 2 that most of current commercialized metal oxide gas sensors aim for indoor air quality control and measure the resistance change caused by the adsorption of total volatile organic compounds (TVOC), where the effect of each kind of gas is not distinguished from the others. Recently, learning algorithms such as artificial neural networks have been applied for classification of the volatile organic compounds from the signals generated by e-nose sensor arrays [11]. Moreover, identification of gas has been explored as a technique to diagnose disease [10] and food quality [127]. CMOS-MEMS monolithic gas sensors have great potential for these applications because of its ability to control the operational states of microhotplates coated with different sensing materials. However, the current challenges remain in developing CMOS-compatible approaches to deposit various sensing materials on the microhotplates. Commercial products require uniform performance, and thus, the discrepancies in morphology and thickness should be minimized.

## 7. Conclusions 

The excellent performance of the MEMS metal oxide semiconductor gas sensor makes it dominant in industrial applications and research projects. As the main structure of a gas sensor to operate the sensing material at an elevated temperature, the microhotplate is fabricated into a membrane or bridge for the sake of thermal isolation to achieve low power consumption. Doped polysilicon and tungsten are recommended as the heater material for operating temperatures below 500 °C and 700 °C, respectively, because of their stability in the temperature range and excellent compatibility with the CMOS process. The temperature uniformity can be improved by optimizing the heater geometry through electro-thermal simulations and validation experiments. Besides the power consumption and stability, other performance parameters such as sensitivity, response/recovery time and selectivity are determined by the sensing material deposited with a post processing approach. Recent research in nanostructured materials shows their enhanced gas sensing performance due to the increase in surface area to volume ratio. CMOS-compatible nanomaterial deposition techniques are still being explored.

Monolithic CMOS-MEMS integration has advantages in size, noise level and power consumption compared to the multi-chip integration. However, temperature constraint of the CMOS circuit must always be kept in mind when designing the MEMS process. The CMOS circuits enable precise control of the on/off state of the microhotplates, which is suitable for sensor arrays with different sensing materials on each unit. In addition, the monolithic integration of the MEMS sensor and CMOS circuit make it possible to develop systems in a package (SiP) or system on a chip (SoC) gas sensing in the future. With a microcontroller unit encapsulated in the same package as the sensor, temperature modulation and signal processing become more efficient because of the reduction in noise during signal transmission.

## Figures and Tables

**Table 1:** Comparison of the four types of gas sensors.

Sensing Principle	Advantages	Disadvantages
Mass	High sensitivity, good reliability, fast response	Piezoelectric substrates are temperature dependent
Optical	High sensitivity, stability over a long lifetime, good selectivity	Difficulty in miniaturization, high cost, high power consumption
Metal oxide (MOX)	Low cost, long lifetime, fast response	Relatively poor selectivity, drift in performance, sensitive to background gas
Thermal (Catalytic)	Low cost, fast response	Detection of flammable gas only, catalyst poisoning, selectivity depends on sensitizers

**Table 2:** Specifications of commercial metal oxide (MOX) gas sensor products.

Company	ams AG			Bosch Sensortec	Figaro	Sensirion
Product No.	iAQ-core P	CCS811	AS-MLV-P2	BME680	TGS8100	SGPC2
Dimension (mm)	15.24 × 17.78	2.7 × 4.0 × 1.1	9.1 × 9.1 × 4.5	3.0 × 3.0 × 0.93	3.2 × 2.5 × 0.99	2.45 × 2.45 × 0.9
Target Gas	CO_2_, TVOC	CO_2_, TVOC	VOC	TVOC	H_2_, C_2_H_5_OH	TVOC
Power consumption/Current	9 mW	1.2–46 mW	34 mW	<0.1 mA in sleep mode	15 mW	1 mA in low power mode
Supply Voltage (V)	3.3	1.8–3.6	2.7	1.2–3.6	1.8	1.62–1.98
Package	SMD	LGA	-	LGA	Ceramic	DFN
Interface	I^2^C	I^2^C	Analog	I^2^C and SPI	-	I^2^C

**Table 3:** Comparison of maximum temperature and complementary metal oxide semiconductor (CMOS) compatibility among various heater materials.

Heater Material	Maximum Temperature (°C)	CMOS Compatibility	Reference
Pt	600	No	[85]
Doped polysilicon	500	Yes	[81]
Ni	300	No	[55]
Mo	800	No	[71]
W	700	Yes	[86]
TiN	700	Yes	[48]

**Table 4:** Performance of miniaturized metal oxide semiconductor gas sensor devices.

Metal Oxide/Sensitizer	Target Gas	Metal Oxide Morphology	Deposition Process	Heater Material/Geometry	Optimal Temperature (°C)	Power Consumption (mW)	Response	Response/Recovery Time (s)	Detection Limit (ppb)	Reference
CuO	H_2_S	Thick film	Drop-cast paste	W/Drive wheel	350	65 @ 600 °C	R_g_/R_a_ = 1.03–1.28 (2–10 ppm)	16.9/49.4	-	[111]
In_2_O_3_	CH_3_CH_2_OH	Thin film	Ink jet printing	Pt/Drive wheel	-	24	R_a_/R_g_ = 2.13 (1 ppm)	-	50	[112]
In_2_O_3_/Au	CH_3_CH_2_OH	Nanofibers	Electro-spinning + paste	Pt/Meander (Coplanar)	140	222.5	R_a_/R_g_ = 13.8 (500 ppm)	12/14	-	[89]
SnO_2_	CH_3_CH_2_OH	Thin film (various grain sizes)	Chemical vapor deposition (CVD) with metal seed layer	Polysilicon/Meander	400	-	R_a_/R_g_ = 2.1~3.1 (90 ppm)	-	-	[113,114]
SnO_2_	CH_3_CH_2_OH	Nanopore array	Hydro-thermal	Pt/Meander	350	30	R_a_/R_g_ = 1.06 (20 ppb)	1/-	20	[115]
SnO_2_	CH_3_CH_2_OH	Nanowire	Hydro-thermal	Pt/Meander (Coplanar)	500	40	R_a_/R_g_ = 26.2 (100 ppm)	1–2/2.5–3.5	-	[90]
SnO_2_	CH_3_CH_2_OH	Thin film	E-beam evaporation	Pt/Meander	400	9	R_a_/R_g_ = 6.5 (300 ppm)	-	-	[73]
SnO_2_	NH_3_, CH_3_CH_2_OH, (CH_2_OH)_2_	Nano-particle	-	Pt/Meander	-	-	R_a_/R_g_ = 6 (100 ppm (CH_2_OH)_2_)	<60/<60	<1000	[116]
SnO_2_/Pt, Pd, Au	CH_3_CH_2_OH CO, H_2_, CH_4_	Thin film	Sputtering	Pt/Meander	300	23 mW (annealing @ 950 °C)	R_a_/R_g_ = 2 (100 ppm CH_3_CH_2_OH)	20–50/10–70	-	[74]
SnO_2_/Pd	CH_3_CH_2_OH	Hollow submicrosphere	Hydro-thermal	Pt/-	300	45	R_a_/R_g_ = ~20 (100 ppm)	1.5/18	-	[117]
SnO_2_/Pd	H_2_	Nanofiber	EHD inkjet printing	W/Meander	185	9.86	R_a_/R_g_ = 8 (2000 ppm)	23/151	-	[118]
SnO_2_/Pt	C_6_H_5_CH_3_ HCHO	Thin film	RF sputtering	Pt/Meander	300–440	31.5 (HCHO) 45 (C_6_H_5_CH_3_)	R_a_/R_g_ = 3.5–4 (HCHO) 3–4 (C_6_H_5_CH_3_)	-	-	[119]
SnO_2_/Au	CH_4_ CO	Thin film	Ion-beam sputtering	Pt/Meander	100 (CO) 250 (CH_4_)	20 (CO) 80 (CH_4_)	-	-	-	[72]
SnO_2_/Sb	CH_3_OH	Porous microsphere	LbL deposition + latex removal	Doped polysilicon/Meander	400	-	R_a_/R_g_ = 40.3 (1 ppm)	-	50	[120]
SnO_2_-CuO	H_2_S	Nanofiber	Electro-spinning	Pt/Meander	200	-	R_a_/R_g_ = 23 (1 ppm)	23/15	<10	[121]
TiO_2_	CO	Thin film	Sputtering	Mo/Double spiral	500	104 (@ 800 °C)	R_a_/R_g_= 6.25 (50 ppm)	0.019/0.034	1000	[71]
TiO_2_	CH_3_OH	Mesoporous film	Hydro-thermal	Polysilicon/Meander	425	-	R_a_/R_g_ = 7 (50 ppm)	4/30	-	[122]
TiO_2_/PdO	H_2_	Thin film (180 + 3 nm)	RF sputtering	Pt/Meander	200	48	-	<10/-		[63]
WO_3_	NO_2_	Porous	Drop-casting	W/Drive wheel	300	65 @ 600 °C	R_g_/R_a_ = 5.25 (100 ppb 50% RH air)	40/205	10	[33]
WO_3_/Au-Pt	H_2_S	Thin film (500 nm)	Sputtering	Pt/Meander	220	-	R_a_/R_g_ = 6.5 (1 ppm)	2/30	-	[123]
WO_3_/Cu_2_O	H_2_S	Nanoneedle	Aerosol-assisted CVD	POCl_3_-doped polysilicon/Double spiral	390	-	R_a_/R_g_ = 27.5 (5 ppm)	2/-	<300	[107]
ZnO	H_2_S	Nanowire	Hydro-thermal	Pt/Drive wheel	300	-	R_a_/R_g_ = 1.78 (200 ppb)	-	5	[124]
ZnO	CH_3_CH_2_OH	Nanowire	Hydro-thermal	N-doped polysilicon/-	400	33	R_a_/R_g_ = 1.6 (809 ppm)	200/~600	-	[97]
ZnO	NH_3_	Nanowire	Hydro-thermo + Dielectrophoretic	W/Drive wheel	350	55 @ 400 °C	R_a_/R_g_ = 4.2 (200 ppm)	228/1290	-	[84]
ZnO/Pd-Ag	CH_4_	Nano-crystalline	Sol-gel spin coating	Ni/Meander	250	120	R_a_/R_g_ = 7.7 (1000 ppm)	8.3/34.8	-	[55]
ZnO-CuO	(CH_3_)_2_CO	Nanoflakes	RF sputtering + thermal oxidation	Ni/Double meander (cavity filled)	300	100 @ 259 °C	R_a_/R_g_ = 0.46 (10 ppm)	22/26	-	[98]
